# *Salmonella* in Birds Migrating through Sweden

**DOI:** 10.3201/eid0906.030072

**Published:** 2003-06

**Authors:** Jorge Hernandez, Jonas Bonnedahl, Jonas Waldenström, Helena Palmgren, Björn Olsen

**Affiliations:** * Research Institute for Zoonotic Ecology and Epidemiology (RIZEE), Färjestaden, Sweden; † Kalmar County Hospital, Kalmar, Sweden; ‡ Umeå University, Umeå, Sweden; § Lund University, Lund, Sweden; ¶ Ottenby Bird Observatory, Degerhamn, Sweden

**Keywords:** Migrating birds, fecal sampling, Salmonella, Letter

**To the Editor**: To determine how common *Salmonella* infection is in the migrating wild bird population, we considered the biology of the bacterium and that of its avian hosts. Previous studies have attempted to determine in which stages wild birds become infected, how infections are acquired, and how this information should be translated into epidemiologic risk assessments for human and animal health. For instance, most published studies originate from small epizootics and are of either dead birds at feeding stations (*1*) or infected birds in or around barns where the livestock has *Salmonella* infection (*2*). This bias has important consequences, as the natural prevalence of *Salmonella* in the non-epizootic situation likely is overestimated. Finding infected birds close to a barn with infected cattle does not prove that transmission occurred from the birds to the animals. In addition, an epizootic at a feeding station does not prove that *Salmonella* normally occurs in the inflicted bird species, as the birds could have became infected through proximity to the infected animals, or in the case of the bird feeder, through feed contaminated from an unknown source. We need baseline surveillance data on the prevalence of *Salmonella* in non-epizootic situations, in healthy bird communities and in different stages of a bird’s life (e.g., during breeding, molting, and migration), to better understand *Salmonella* epidemiology in relation to wild birds.

We focused on the migratory bird fauna of the North Western Palearctic, where most of the birds migrate south to spend the nonbreeding season in continental Europe and Africa. In these areas, certain species, such as gulls, corvids, starlings, and thrushes, may overwinter in agricultural and urban areas were domestic animals are present. We sampled apparently healthy birds trapped on active migration at Ottenby Bird Observatory (56°12'N, 16°24'E), on the southernmost tip of the island Öland, southeast Sweden, during the migration periods July–November 2001, March–May 2002, and July–December 2002. We used a standardized trapping and sampling scheme, previously used at the same site for large-scale screening of *Campylobacter* infections in wild birds (*3*). To apply a random procedure in selection of species and persons, every 10th bird banded during the migration periods was sampled for *Salmonella* spp*.* We did not sample recaptured birds previously banded by us.

In total, 2,377 samples from 110 species of migratory birds (1,086 samples in autumn 2001, 777 in spring 2002, and 514 in autumn 2002) were analyzed for *Salmonella* infections. We applied routine procedures for isolation of putative *Salmonella* isolates, with enrichment in Rappaport-Vassiliadis broth and injection into Xylose-Lysine-Desoxycholate (XLD) agar. On this media, most *Salmonella enterica* serotype Enterica appears as red transparent colonies with black centers. Colonies with growth characteristics of *Salmonella* were observed in 236 samples, and full phenotypic identification was performed on these isolates by using standard biochemical and serologic testing. By using the API system (*4*), the isolates were identified as *Citrobacter youngae*, *C. braakii, C. freundii, Escherichia vulneris, E. coli, Hafnia alvei, Klebsiella pneumoniae ozaenae, Acinetobacter baumanii , Providencia stuartii/rettgeri*, and *Yersinia kristen senii.* Only one of the isolates, obtained from a Mistle Thrush (*Turdus viscivorus*) and sampled during the spring migration 2002, carried *Salmonella.* This isolate was characterized by serotyping according to the Kauffman-White serotyping scheme (*5*) at the reference laboratory of the Swedish Institute for Infectious Disease Control. The thrush isolate was identified as *S.* Schleissheim, a rare *Salmonella* serotype. Human salmonellosis caused by this serotype has been previously reported only in Turkey (*6*). No reservoir of *S.* Schleissheim, in animals or in humans, has been reported in Sweden in the last 10 years covered by the current epidemiologic records.

The failure to find *Salmonella* was probably not caused by technical problems. The sampling methods used, with fecal samples from fresh droppings or cloacal swabs, are well-established techniques for studying *Salmonella* prevalence in birds (*2*,*7*,*8*). The laboratory methods used, with enrichment in Rappaport-Vassaliadis broth and subsequent culturing on XLD-agar, are extremely sensitive for detecting *Salmonella*, even for samples highly contaminated with other *Enterobacteriaceae* (*9*). Thus, in this large dataset, only one *Salmonella* isolate was found, representing a serotype rarely observed in clinical or veterinary samples. In particular, one serotype, *S.* Typhimurium DT40, has been associated with epizootics in wintering passerine birds (*10*), but this serotype was not found in any of our samples.

Results from our study indicate that the prevalence of this serotype in the healthy wild bird population is low. Our dataset was composed of many different species, but the number of tested individual birds for each species was low in many cases. Earlier studies have pointed to certain species (gulls and corvids) in which the prevalence of *Salmonella* is sometimes high (2% to 20%), and argued that concern should be strong about epidemiologic disease transmission with these birds (*7*,*8*). These species have the capability to live in an opportunistic manner in close proximity to humans and can base their diet on waste products and garbage. Most bird species, however, have little or no niche overlap with humans or domesticated animals; virtually no data exists on the occurrence of *Salmonella* in this major group of migrating birds during a non-epizootic situation. Our results suggest that the natural occurrence of *Salmonella* in healthy birds during migration in Sweden may be low. Therefore, the *Salmonella* incidence is probably also low for most wild bird species. We suggest that researchers consider analyzing the non-epizootic natural occurrence of *Salmonella* in wild birds. Accumulated knowledge from many different regions, over many years, is a prerequisite for thorough risk assessment of the importance of *Salmonella* carriage in wild birds.
